# Hunger as a Test of Poverty

**Published:** 1895-11-16

**Authors:** 


### Hunger as a Test of Poverty.

The feeding of schoolchildren is a difficult problem. The chance of a free meal may lead to home starvation, while, if money is exacted, what is given may be spent in other ways, for a child's longing is not always for plain food. Again, it is most difficult to decide in which case help should be offered, for the appearance of a child as to dress and other matters is often a by no means trustworthy index of the condition of the larder in its home. If there is one thing of more importance than another in the report of the sub-committee appointed by the London School Board to consider this subject, it is the attention drawn to the fact that, in the present condition of affairs among the poor of London, food is about the cheapest thing they buy. Rent and clothes on the other hand are dear, and it happens in endless cases that the poverty-stricken looking child, whose clothes are a disgrace, and whose home (!) is but an overcrowded and insanitary den, may yet be having food enough —such as it is. The committee then suggest that free meals should not be an end in themselves, but that a continuous record should be kept of their recipients, so that search may be made as to the home surroundings of those who apply again and again. On the one hand it is not impossible that in many of these cases the parents may be at fault, and that the proper agency to deal with them may be the Society for the Prevention of Cruelty to Children, rather than that for the giving of free meals; while in other cases the real want may be help at home, relief in sickness, or assistance in getting work. Again, then, we come back to the old moral, that clothes are no criterion of condition, and that the real way to help the poor is not alone by money, but by personal service in the investigation of each individual case.

